# Coping and Positive Body Image in Young Women with Breast Cancer: The Buffering Role of Social Support

**DOI:** 10.3390/healthcare13030346

**Published:** 2025-02-06

**Authors:** Márcia Almeida, Maria Inês Griff, Tânia Brandão

**Affiliations:** 1 School of Psychology, Ispa—Instituto Universitário, Rua Jardim do Tabaco, 44, 1149-041 Lisboa, Portugal; marciaalexandra2001@hotmail.com (M.A.); inesgriff@gmail.com (M.I.G.); 2 William James Center for Research, Ispa—Instituto Universitário, Rua Jardim do Tabaco, 44, 1149-041 Lisboa, Portugal

**Keywords:** coping strategies, positive body image, perceived social support, breast cancer

## Abstract

**Background/Objectives**: Considering the unique body image challenges faced by young women with breast cancer, this study aims to deepen our understanding of how coping strategies and perceived social support contribute to fostering positive body image in this population. This is an important issue as body image is capable of influencing women’s self-esteem and psychological adaptation to breast cancer. **Methods**: A sample of 157 young women with breast cancer (M age = 41.43; SD = 6.05; Min 26 years, Max 50 years), 43% of whom had undergone breast reconstruction, completed an online survey. The survey included measures of coping strategies, positive body image, and perceived social support. **Results**: No significant differences in coping strategies, perceived social support, or positive body image were observed based on reconstruction status. Moderation analyses revealed that both avoidant and problem-focused coping were significantly associated with positive body image at moderate and high levels of perceived social support, but not at low levels of perceived social support. Perceived social support moderated the relationship between coping strategies and positive body image in women with breast cancer. **Conclusions**: These findings highlight the importance of social support in enhancing the effectiveness of both avoidant and problem-focused coping strategies in promoting positive body image. Future interventions should prioritize strengthening social support networks to optimize psychological outcomes in this population.

## 1. Introduction

### 1.1. Body Image in Breast Cancer

In 2022, breast cancer (BC) was the most frequently diagnosed cancer among women globally, accounting for approximately 12% of all new cancer cases reported annually [1]. While there is no universally accepted age threshold to define young women with BC, studies often use menopause age (>55 years) as a dividing line to classify women with BC into younger and older groups, with significant clinical implications [2]. Although BC is less prevalent among younger women, they often face more aggressive tumor biology compared to older patients, leading to increased complications, worse outcomes, reduced curability, and poorer prognoses. This disparity is further aggravated by the fact that their cancers are commonly diagnosed at more advanced stages, as younger women are typically excluded from routine screening programs [3].

While a BC diagnosis at any age is profoundly disruptive, significantly impacting psychological functioning and quality of life (e.g., anxiety, depression, pain) [4], young women face distinct challenges. These challenges encompass reproductive health concerns, parenting responsibilities, body image disturbances, and enduring psychosocial effects, such as heightened fear of cancer recurrence and problems in interpersonal relationships, all of which contribute to the complexity of their experiences [5,6].

Body image holds particular significance for young women with BC, as cancer treatments are likely to impact body image [7]. Also, younger women often experience lower body appreciation, increased sexual objectification, and heightened pressures from social and cultural standards compared to older women [8]. Body image disturbance is a prevalent concern among individuals who have survived BC, being more prevalent among young survivors, and being associated with psychological adaptation (e.g., emotional distress and psychological well-being) [7].

Recent research has increasingly shifted its focus toward recognizing the importance of positive body image, as it is a dimension linked to self-esteem and psychological well-being [8,9]. It can be defined as “an overarching love and respect for the body, which allows appreciation of appearance and function and an awareness of the body’s needs” (p. 122 [10]). It is a construct different from negative body image, which includes dissatisfaction or concerns related to appearance [11]. Some researchers emphasize two dimensions: body appreciation (including acceptance, approval, and respect for one’s body, and an acknowledgment of its needs) and body image flexibility (i.e., the ability to manage thoughts and emotions about one’s body) [8].

In the context of BC, body image has been approached from both positive and negative perspectives, examining aspects such as satisfaction with body shape and distress caused by hair loss [12,13]. Additionally, qualitative studies with women with BC have identified key dimensions of positive body image, including body appreciation, acceptance of altered appearance, body confidence, and the perceived importance of a healthy body [12,14].

For BC patients, a positive body image is often crucial in the recovery process, as it helps reduce depressive symptoms and enhances psychological adjustment [14,15]. The development of a positive body image may be influenced by personal and external factors, such as coping strategies and perceived social support, which help women navigate the challenges associated with physical changes and emotional stress during cancer treatment (e.g., [14]), as described as follow.

### 1.2. The Role of Coping Strategies

Coping strategies are central to understanding how individuals manage the psychological and emotional challenges associated with breast cancer [16]. This study is guided by the Stress Appraisal and Coping Model developed by Lazarus and Folkman [17], which conceptualizes coping as “cognitive and behavioral efforts to manage specific external and/or internal demands that are appraised as taxing or exceeding the resources of the person” (p. 141 [17]). Three primary coping styles have been extensively studied: problem-focused (actively addressing the situation), emotion-focused (managing emotional responses), and avoidant coping (distancing or disengaging from the situation) [17].

In the context of cancer, coping plays a significant role in influencing adaptation and overall well-being [16,18]. Avoidant coping is often linked to heightened psychological distress and challenges in adapting to the disease, while engagement coping shows inconsistent relationships with distress outcomes [16,18,19]. Some authors emphasize that negative coping strategies, such as avoidant coping, are not always detrimental to the adaptation process, as their effectiveness can vary depending on the context and individual characteristics [18].

Coping in the context of body-image stressors is an essential area of focus, allowing the employment of strategies to deal with thoughts, feelings, and situations resulting from anything from treats to body image [20]. However, research on the role of coping strategies in body image outcomes remains limited, particularly in the context of BC. However, Zhu et al. [3] provide valuable insights into these associations. Their study revealed that higher levels of avoidant coping were linked to greater body image disturbance, while a more confrontational coping style was associated with reduced body image disturbance. Interestingly, acceptance or resignation—characterized by indifference and disinterest—was also found to be associated with higher levels of body image disturbance.

### 1.3. The Buffer Role of Social Support

Perceived social support is typically understood as an individual’s perception of the availability and adequacy of emotional, instrumental, and informational assistance from significant sources, such as family, friends, and other key individuals [21]. Higher perceived social support is associated with better emotional health and resilience, emphasizing its role in promoting adaptive coping and mitigating distress in challenging contexts like chronic illness, e.g., [22]. According to the Social Support Buffer Theory [23], perception of available support can protect individuals from the harmful impact of stress by mitigating their stress reactions.

Among BC patients, social support has been associated with improved quality of life, enhanced global health status, reduced fear of cancer recurrence, and better adjustment trajectories over time [24,25,26,27]. Additionally, social support also plays a significant role in fostering greater satisfaction with body image, and in dealing with changes in body image cognition after surgery [28,29].

Social support and coping appear to be closely linked. A review by Bottaro et al. [30] highlighted a bidirectional association, consistent across various cancer types. Specifically, higher perceived social support was correlated with greater use of adaptive coping strategies, such as approach and appraisal coping and problem-solving, while being associated with lower levels of avoidant coping. Conversely, the adoption of adaptive coping mechanisms positively influenced levels of perceived social support, creating a reinforcing cycle of effective coping and support [30].

Cohee et al. [31] emphasize that experiencing social constraints, particularly from partners, can contribute to the use of avoidant coping strategies. Limited or restrictive communication and lack of understanding within social interactions might exacerbate emotional withdrawal, leading individuals to cope by avoiding stressors rather than addressing them directly. Furthermore, the presence of a strong social network has been linked to better health outcomes, as social support can influence coping strategies, enabling patients to manage stress more effectively [32].

### 1.4. The Present Study

Body image is a significant concern for young women in general and young women with BC in particular [7,8]. While previous research on body image in the context of cancer has predominantly focused on body image difficulties and the negative impacts of cancer, limited attention has been paid to the concept of positive body image within this population. This gap often neglects the potential for individuals to develop resilience, body acceptance, or appreciation despite the adversities associated with cancer [12,14]. Exploring positive body image is crucial, as it represents a vital dimension that can significantly influence women’s self-esteem and psychological well-being [9,10].

Guided by the Stress Appraisal and Coping Model [17], which emphasizes the role of cognitive appraisal and coping strategies in managing stress, this study investigates the role of coping strategies in fostering positive body image. While some previous research has shown that avoidant coping strategies are often associated with more negative outcomes, including greater body image problems [3], the relationship between coping strategies and positive body image remains unexplored. Furthermore, studies examining engagement coping strategies have yielded inconsistent findings regarding their effectiveness in the context of cancer, suggesting that the relationship between coping strategies and outcomes, including body image, may be more nuanced and complex than previously understood. This study aims to address these gaps by shedding light on the role of coping strategies in fostering positive body image.

**Figure 1:**
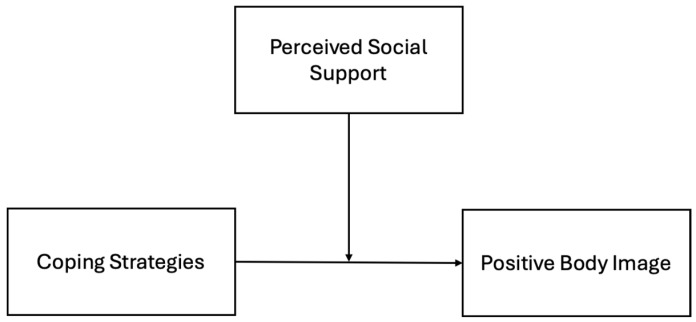
Conceptual model.

Additionally, guided by the Social Support Buffer Theory [23], which emphasizes the protective role of social support in mitigating the adverse psychological and physical effects of stress, this study aimed to address an important gap in the literature by exploring the moderating role of perceived social support in the relationship between coping strategies and positive body image (see Figure 1 for the conceptual model proposed). While prior research has examined the general benefits of social support and its impact on mental health outcomes, in the context of cancer [24,25,26,27], little is known about how social support interacts with coping strategies to foster positive body image in young women with BC.

Specifically, our research hypotheses are as follows:

**H1.**  *Avoidant coping strategies are negatively associated with positive body image, whereas problem-focused coping strategies are positively associated with positive body image in young women with BC.*

**H2.**  *Perceived social support moderates the relationship between coping strategies and positive body image. Specifically, higher perceived social support buffers against the negative impact of avoidant coping strategies and amplifies the positive impact of problem-focused coping strategies on positive body image.*

By elucidating these relationships, the study aims to provide insights that can inform psychosocial interventions to promote well-being and psychological resilience among young women diagnosed with BC.

## 2. Materials and Methods

### 2.1. Participants

Participants were recruited between February and May 2024 based on the inclusion criteria of (a) identifying as female, (b) being between 20 and 50 years old, (c) having a BC diagnosis, and (d) having experienced mastectomy as part of treatment. The sample size was determined using G*Power (version 3.1), based on a medium effect size (f^2^ = 0.15), an alpha error probability of 0.05, a power of 0.90, and five predictors (social support, two types of coping, and two interaction terms), resulting in a required total sample size of 116 participants.

A total of 162 participants accessed the link; three did not consent to participate, and two consented but reported an age above 50, and thus they were excluded from the study. The final study sample included 157 women, aged between 26 and 50 years (M = 41.43; SD = 6.05), who had been diagnosed with BC and undergone mastectomy. Most participants were Portuguese (98.1%), with a small percentage identifying as Brazilian (1.9%). In terms of education, 43.9% had completed an undergraduate degree, 22.3% held a master’s degree, and smaller proportions had completed high school or doctoral-level studies. Most participants (78.3%) were professionally active at the time of the study. The sample was predominantly composed of individuals in a relationship (81.5%), and 68.8% of the women had children, with an average of two children (37.6%). The majority reported a middle-income level (69.4%), and the average household size was four people.

Participants’ clinical information revealed that 40.1% detected their BC through self-examination, followed by 28% who noticed specific symptoms, and 26.8% who identified the cancer during a routine medical checkup. The average age at diagnosis was 39.86 years (SD = 5.91). For 98.1% of participants, this was their first BC diagnosis, with 63.1% reporting no family history of BC and 76.4% without other comorbid conditions.

Regarding treatment, 98.1% of participants had initiated treatment, with the majority undergoing chemotherapy (86.6%) and mastectomy (66.2%). Additionally, 58.6% received hormone therapy, while 56.1% underwent radiotherapy. Most women (65%) received chemotherapy prior to surgery, while 35.7% received it afterward. At the time of the study, 65.6% were no longer undergoing treatment, although 80.9% were continuing hormone therapy. Regarding breast reconstruction post-mastectomy, 42.7% had undergone reconstruction, with 34.4% having it performed either immediately after or during mastectomy. For those who did not undergo immediate reconstruction, the average wait time was approximately four months (M = 4.00; SD = 8.94).

### 2.2. Measures

#### 2.2.1. Positive Body Image

Positive body image was assessed using the Body Appreciation Scale-2 (BAS-2), a unidimensional scale developed by Tylka and Wood-Barcalow [33] and validated for the Portuguese population by Lemoine et al. [34]. The BAS-2 consists of 10 items, such as “I respect my body” and “I feel good about my body”, rated on a 5-point Likert scale ranging from 1 (never) to 5 (always). Higher scores reflect greater body appreciation. In the current study, Cronbach’s alpha was 0.95.

#### 2.2.2. Coping Strategies

Coping strategies were assessed using the Brief COPE, developed by Carver [35] and adapted for Portuguese samples by Pais-Ribeiro et al. [36]. This instrument evaluates coping responses to stress and consists of 28 items distributed across 14 subscales, such as “I’ve been taking action to try to make the situation better” and “I’ve been saying to myself ‘this isn’t real’”. Each item is rated on a 4-point Likert scale ranging from 0 (“I haven’t been doing this at all”) to 3 (“I’ve been doing this a lot”).

For the purposes of this study, two types of coping were measured: (1) problem-focused coping (which included active coping, use of informational support, and positive reframing), with high scores on this dimension indicating coping strategies aimed at actively changing or addressing the stressful situation, and (2) avoidant coping (which included denial, substance use, and behavioral disengagement), with high scores indicating physical or cognitive efforts to disengage from the stressor. In the current study, Cronbach’s alpha was 0.83 for problem-focused coping and 0.73 for avoidant coping.

#### 2.2.3. Perceived Social Support

Perceived social support was measured using the Multidimensional Scale of Perceived Social Support (MSPSS), developed by Zimet et al. [21] and adapted for the Portuguese population by Carvalho et al. [37]. The MSPSS assesses support from three sources: family, friends, and significant others. The scale comprises 12 items, such as “My family really tries to help me” and “I have a special person who is there when I need them”, rated on a 7-point Likert scale ranging from 1 (very strongly disagree) to 7 (very strongly agree). Higher scores reflect more perceived social support. In the current study, for the total score, Cronbach’s alpha was 0.95.

### 2.3. Procedure

The study received ethical approval from the Ethics Committee of Ispa—Instituto Universitário (protocol code I-134-12-23; date of approval—15 December 2023). Following approval, participants were recruited through multiple channels, including breast cancer support organizations in Portugal, such as the Portuguese Association for Support to Women with Breast Cancer, and social media groups and networks. Recruitment materials included information about the study’s purpose, confidentiality assurances, and a link to the Google Forms questionnaire.

Prior to participation, individuals provided informed consent electronically, confirming their age to be between 20 and 50 years, and their willingness to engage in the study. The survey began with sociodemographic questions, followed by three psychometric instruments: the BAS-2, the MSPSS, and the Brief COPE. Anonymity and confidentiality of responses were ensured, and participants were explicitly informed that their data would be used exclusively for academic and scientific purposes.

To address potential discomfort or distress arising during survey completion, participants were provided with contact information for free psychological support services. This ensured that ethical standards for participant care and well-being were maintained throughout the study.

### 2.4. Data Analysis

Descriptive analyses were conducted to summarize the means and standard deviations of the study variables, including avoidant coping, problem-focused coping, positive body image, and perceived social support. Pearson correlation coefficients were calculated to examine the relationships among these variables, providing an initial understanding of the associations between coping strategies, perceived social support, and positive body image. Considering the focus of this study on body image, we examine potential differences in study variables based on whether participants underwent breast reconstruction. For this purpose, *t*-tests were conducted with independent samples. All these analyses were performed in SPSS (Version 26).

Moderation analyses were subsequently conducted using the PROCESS macro for SPSS (Version 4.2) [38] to examine whether perceived social support moderated the relationships between avoidant coping and positive body image, as well as between problem-focused coping and positive body image. The moderation analysis was conducted using 5000 bootstrap samples with a 95% confidence interval. Conditional effects were examined at three levels of perceived social support: one standard deviation (−1 SD) below the mean (low support), the mean (moderate support), and one standard deviation (+1 SD) above the mean (high support).

For each moderation analysis, the model summary provided the explained variance (*R*^2^) and significance levels for the interaction terms. Conditional effects were used to assess the relationship between the coping strategies and positive body image at different levels of perceived social support. Statistical significance was determined at *p* < 0.05. Scatterplots were generated to visually represent the interaction effects, highlighting how the relationship between coping strategies and positive body image changes depended on levels of perceived social support.

## 3. Results

### 3.1. Descriptive Statistics and Correlations

Table 1 presents the descriptive statistics (mean and standard deviation) and correlation coefficients for the study variables. Avoidant coping was positively and significantly correlated with problem-focused coping, positive body image, and perceived social support. Problem-focused coping was also positively and significantly correlated with positive body image and perceived social support. Positive body image demonstrated a significant positive correlation with perceived social support.

### 3.2. Differential Analyses

No significant differences were observed in avoidant coping (*t*(155) = 0.90, *p* = 0.185) or problem-focused coping (*t*(155) = 0.52, *p* = 0.303) between participants who underwent breast reconstruction and those who did not. Similarly, there were no statistically significant differences in positive body image (*t*(155) = 1.82, *p* = 0.070) or perceived social support (*t*(118.633) = 1.79, *p* = 0.075) based on breast reconstruction status.

### 3.3. Moderation Analyses

A moderation analysis was conducted to examine whether perceived social support moderated the relationship between coping and positive body image. Two moderation models were tested: one for avoidant coping and one for problem-focused coping.

#### 3.3.1. Avoidant Coping

For the avoidant coping, the overall model was statistically significant (*F*(3, 153) = 27.88, *p* < 0.001), explaining 35% of the variance in positive body image (*R*^2^ = 35.34). The interaction term (avoidant coping × perceived social support) contributed significantly to the model (*R*^2^ change = 0.03, *F* = 7.39, *p* < 0.01), indicating a moderating effect of perceived social support.

The analysis revealed that avoidant coping had a significant negative main effect on positive body image, suggesting that higher levels of avoidant coping were associated with lower levels of positive body image. In contrast, perceived social support did not exhibit a significant main effect (see Table 2).

**Figure 2:**
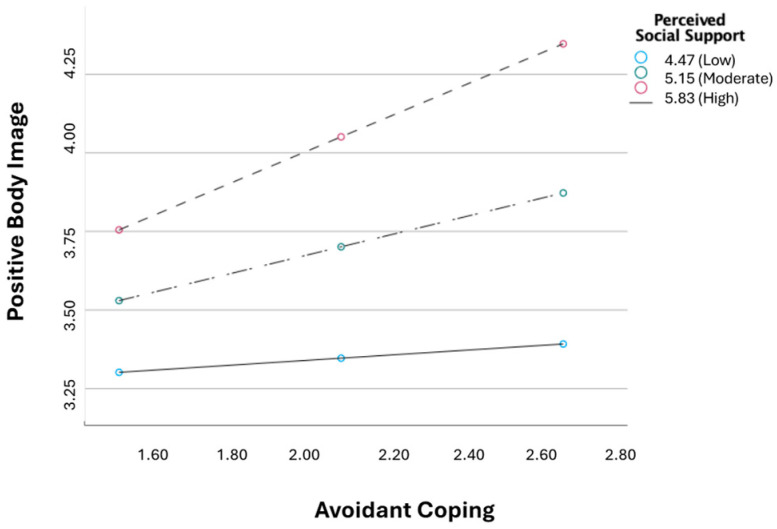
Interaction between avoidant coping and perceived social support in explaining positive body image.

The interaction effect between avoidant coping and perceived social support was significant (*b* = 0.32, *p* < 0.01). This indicates that the effect of avoidant coping on positive body image depends on levels of perceived social support. The conditional effects of avoidant coping on positive body image were examined at three levels of perceived social support (1 SD below the mean = low perceived social support; mean = moderate perceived social support; and 1 SD above the mean = high perceived social support). Avoidant coping was significantly associated with positive body image only for the moderate (*b* = 0.30, *p* < 0.001) and the high perceived social support (*b* = 0.52, *p* < 0.001). No significant associations were found for the low perceived social support (*b* = 0.08, *p* = 0.491) (see Figure 2).

#### 3.3.2. Problem-Focused Coping

For the problem-focused coping, the overall model was statistically significant (*F*(3, 153) = 27.75, *p* < 0.001) and explained 35% of the variance in positive body image (*R*^2^ = 35.24). The interaction term (problem-focused coping × perceived social support) contributed significantly to the model (*R*^2^ change = 0.03, *F* = 6.71, *p* < 0.05), indicating a moderating effect of social support.

Problem-focused coping had a significant negative main effect on positive body image, suggesting that higher levels of problem-focused coping were associated with lower levels of positive body image. Perceived social support did not exhibit a significant main effect (see Table 3).

**Figure 3:**
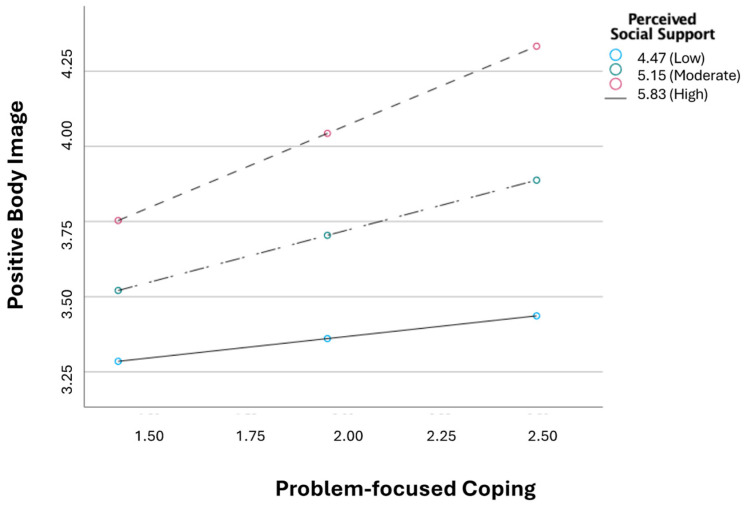
Interaction between problem-focused coping and perceived social support in explaining positive body image.

The interaction between problem-focused coping and perceived social support was statistically significant (*b* = 0.29, *p* < 0.05). This indicates that the effect of problem-focused coping on positive body image depends on the level of perceived social support. Again, the conditional effects of problem-focused coping on positive body image were examined at three levels of perceived social support (1 SD below the mean = low perceived social support; mean = moderate perceived social support; and 1 SD above the mean = high perceived social support). Problem-focused coping was only significantly associated with positive body image for moderate perceived social support (*b* = 0.34, *p* < 0.001) and high perceived social support (*b* = 0.54, *p* < 0.001). No significant associations were found for low perceived social support (*b* = 0.14, *p* = 0.217) (see Figure 3).

## 4. Discussion

The present study examined the influence of coping strategies and perceived social support on positive body image among young women with BC, with a particular focus on the moderating role of social support in this relationship. Specifically, it investigated how social support acts as a buffer, potentially mitigating the negative effects of maladaptive coping strategies, and enhancing the positive impact of adaptive coping mechanisms on improving positive body image. Overall, findings suggested that both avoidance and problem-focused coping strategies can be useful in promoting a positive body image, but only for those with moderate/higher levels of perceived social support.

The finding that greater reliance on avoidant coping strategies is associated with lower levels of positive body image is consistent with existing literature and our H1, emphasizing the detrimental psychological effects of avoidance-based approaches for emotional distress and, particularly, body image issues [3,31]. Avoidant coping, characterized by disengagement, denial, or distraction, often reflects a lack of active engagement with stressors or challenges [18]. This can include those related to body image concerns. Such strategies may hinder the emotional processing necessary for adapting to the physical changes and psychosocial impacts of BC [18]. As found by Zhu et al. [3] higher levels of avoidant coping were linked to greater body image disturbance, supporting the negative link between avoidant approaches and body image outcomes.

However, the results suggested that when moderate or high levels of perceived social support were present, the negative impact of avoidant coping on positive body image may be mitigated, as avoidant coping was associated with more positive body image at moderate and high levels of social support, partially supporting our H2. This result suggests that avoidant coping is not always harmful, and its outcomes can vary based on the situation and the individual’s resources (e.g., whether they have social support) [19]. Social support is essential in mitigating distress in challenging contexts, like chronic illness e.g., [22]. The perception of social support appears to play a critical role in fostering a positive body image in the context of BC. Social support may provide emotional, informational, and practical resources that help offset the adverse effects often linked to avoidant coping. Previous research highlights that social constraints can lead to increased use of avoidant coping, which is typically associated with negative mental health outcomes [39]. However, our findings suggest that individuals employing avoidant coping may still preserve or improve their positive body image when they receive reassurance, encouragement, or practical assistance from their support network. Also, moderate and high levels of perceived social support may introduce positive reinforcement, affirmations, or adaptive coping alternatives, subtly guiding individuals toward a more positive self-view, even if they continue to engage in avoidant behaviors [28,29]. This highlights the importance of social support in mitigating the potential downsides of avoidant coping strategies. It is important to note, however, that evidence from patients with cancer suggests a bidirectional relationship between perceived social support and body image. Specifically, individuals experiencing less body image distress are more capable of activating and utilizing available social support resources [40,41]. This highlights the complex interplay between perceived social support and body image, suggesting that interventions aimed at improving body image may also enhance individuals’ ability to seek and utilize social support, and vice versa. Addressing both aspects in psychosocial interventions could create a reinforcing cycle that promotes overall psychological well-being in individuals with cancer.

Our findings regarding problem-focused coping suggest a complex relationship with positive body image among young women with BC. Specifically, higher levels of problem-focused coping were associated with lower levels of positive body image, which ran contrary to our expectations, as this type of coping is usually linked to positive outcomes e.g., [3]. It is possible that problem-focused coping, which involves actively addressing stressors [18], may have limited effectiveness when dealing with body image concerns in this population. Many of the stressors related to body image, such as physical changes due to BC and its treatment, may not have immediate or tangible solutions. This inability to “fix” the perceived problems could lead to frustration or even a heightened focus on physical imperfections, which ultimately diminishes positive body image. Indeed, research on body image coping has shown that coping by appearance fixing tends to lead to poor body image [42].

Importantly, the moderation analyses revealed a more nuanced picture. At moderate or higher levels of perceived social support, problem-focused coping was positively associated with positive body image. This finding highlights the critical role of social support in enhancing the effectiveness of problem-focused coping for body image concerns. When individuals receive reassurance, encouragement, and practical assistance from their support networks, their problem-focused efforts may become more effective in addressing and reframing body image challenges. Social support appears to buffer against the potential negative effects of problem-focused coping by fostering a sense of connection, providing external resources, and reducing feelings of isolation. Conversely, when social support is low, problem-focused coping may exacerbate feelings of overwhelm and isolation. In such cases, the lack of external validation and assistance might make individuals feel less capable of managing the stressors tied to body image, further contributing to a decline in their positive body image.

### 4.1. Limitations and Future Research

While this study provides valuable insights into the relationship between coping strategies, perceived social support, and positive body image among young women with BC, some limitations should be considered. First, the cross-sectional nature of the design limits our ability to draw causal inferences regarding the relationships between coping strategies, perceived social support, and positive body image. Future research employing longitudinal or experimental designs is needed to establish causal pathways and capture the dynamics of these relationships over time.

Second, the sample characteristics and generalizability warrant consideration, and the potential for selection effects should be noted. Participants were recruited online, primarily through the social media platforms of the Portuguese Association for Support to Women with Breast Cancer, which may have introduced selection bias. Individuals actively engaged with such networks may have higher levels of perceived social support and access to resources, which could limit the applicability of the findings to women with breast cancer who are less active online or lack access to these platforms. Additionally, the sample may not fully represent diverse sociodemographic or cultural backgrounds, further constraining generalizability. Thus, future research should adopt a more diverse recruitment strategy, including participants from different settings (including in-person recruitment through hospitals or clinics). Also, expanding the sample size and including diverse populations (e.g., different socio-economic backgrounds or cancer subtypes and different stages of the disease) would enhance the applicability of the findings.

Fourth, as this study relied on social media for recruitment, we were unable to document recruitment success or response rates due to the lack of access to data on the total number of individuals who viewed the study invitation. Future studies could consider alternative recruitment strategies, such as targeted advertisements with engagement metrics or collaborations with organizations that track participant outreach, to better estimate response rates and recruitment success. Fifth, we could not independently verify participants’ clinical status, which may introduce some degree of misclassification bias. Future studies should consider implementing additional eligibility verification measures, such as requesting medical confirmation or recruiting exclusively through healthcare institutions, to minimize potential misclassification bias.

Finally, measurement considerations must be addressed. While validated scales were used to assess key variables, self-reported measures are inherently susceptible to biases such as social desirability or recall errors.

### 4.2. Practical Implications

Healthcare providers working with young women undergoing BC treatment should consider incorporating both coping strategies and social support systems into care plans to improve body image outcomes.

Healthcare providers and counselors should focus on creating tailored interventions that include both social support and coping strategies. Encouraging the development of moderate to high levels of perceived social support can enhance the effectiveness of various coping mechanisms, improving body image in young women with BC. Offering group support sessions that foster peer interactions can increase social support, which in turn may help mitigate the negative effects of avoidant coping and enhance positive body image outcomes. These sessions can help patients share experiences and coping strategies, promoting a supportive environment.

Incorporating both emotional and practical support elements into cancer care programs ensures that patients have access to comprehensive support, which enhances their ability to adopt effective coping strategies or at least buffer against the effects of less adaptive coping strategies.

## 5. Conclusions

Overall, our findings underscore the complexity of coping processes and highlight the importance of external resources like social support in shaping health outcomes. They suggest that interventions aimed at fostering robust social support networks could benefit individuals who rely on avoidant coping strategies, potentially improving their body image and overall psychological well-being.

## Figures and Tables

**Table 1:** Descriptive statistics and correlations among study variables (N = 157).

	M (SD)	1.	2.	3.	4.
1. Avoidant coping	2.08 (0.57)	-			
2. Problem-focused coping	1.96 (0.54)	0.91 *	-		
3. Positive body image	3.75 (0.67)	0.41 *	0.42 *	-	
4. Perceived social support	5.15 (0.69)	0.37 *	0.40 *	0.52 *	-

* *p* < 0.001.

**Table 2:** Results from moderation analysis for avoidance coping (N = 157).

	Coeff	SE	*t*	*p*	LL95%CI	UL95%CI
Main effects						
Avoidant coping	−1.36	0.62	−2.21	<0.005	−2.58	−0.15
Perceived social support	−0.16	0.22	−0.71	0.477	−0.60	0.28
Interaction term	0.32	0.12	2.72	<0.01	0.09	0.56
Conditional effects						
−1 SD	0.08	0.12	0.69	0.491	−0.15	0.31
Mean	0.30	0.08	3.64	<0.001	0.14	0.47
+1 SD	0.52	0.12	4.45	<0.001	0.29	0.75

**Table 3:** Results from moderation analysis for problem-focused coping (N = 157).

	Coeff	SE	*t*	*p*	LL95%CI	UL95%CI
Main effects						
Problem-focused coping	−1.16	0.58	−2.00	<0.05	−2.30	−0.02
Perceived social support	−0.07	0.20	−0.36	0.719	−0.46	0.32
Interaction term	0.29	0.11	2.59	<0.05	0.07	0.51
Conditional effects						
−1 SD	0.14	0.11	1.24	0.217	−0.08	0.36
Mean	0.34	0.09	3.82	<0.001	0.16	0.52
+1 SD	0.54	0.12	4.42	<0.001	0.30	0.78

## Data Availability

Data are unavailable due to privacy or ethical restrictions.

## References

[1] Bray F., Laversanne M., Sung H., Ferlay J., Siegel R.L., Soerjomataram I., Jemal A. (2024). Global cancer statistics 2022: GLOBOCAN estimates of incidence and mortality worldwide for 36 cancers in 185 countries. CA Cancer J. Clin..

[2] Shah A.N., Carroll K.J., Gerratana L., Lin C., Davis A.A., Zhang Q., Cristofanilli M. (2021). Circulating tumor cells, circulating tumor DNA, and disease characteristics in young women with metastatic breast cancer. Breast Cancer Res. Treat..

[3] Zhu F., Liu C., Zhang W., Qiang W., Yin X., Lu Q. (2023). The mediating effect of coping styles between self-compassion and body image disturbance in young breast cancer survivors: A cross-sectional study. BMC Nurs..

[4] Graham J. (2024). Breast cancer: The psychological impact of diagnosis, treatment, and remission. Cureus.

[5] Martino M.L., Lemmo D., Gargiulo A. (2021). A review of psychological impact of breast cancer in women below 50 years old. Health Care Women Int..

[6] Vuong S., Warner E. (2024). Breast cancer survivorship among younger patients: Challenges and opportunities—Narrative review. Ann. Palliat. Med..

[7] Thakur M., Sharma R., Mishra A.K., Gupta B. (2022). Body image disturbances among breast cancer survivors: A narrative review of prevalence and correlates. Cancer Res. Stat. Treat..

[8] Modica C.A., Markley A.E. (2024). Reciprocal longitudinal associations between positive body image and wellbeing among early-adult women. Body Image.

[9] Linardon J., Anderson C., McClure Z. (2023). Body appreciation predicts better mental health and wellbeing. A short-term prospective study. Body Image.

[10] Wood-Barcalow N.L., Tylka T.L., Augustus-Horvath C.L. (2010). “But I Like My Body”: Positive body image characteristics and a holistic model for young-adult women. Body Image.

[11] Thompson J.K., Coovert M.D., Stormer S.M. (1999). Body image, social comparison, and eating disturbance: A covariance structure modeling investigation. Int. J. Eat. Disord..

[12] Grogan S., Mechan J. (2017). Body image after mastectomy: A thematic analysis of younger women’s written accounts. J. Health Psychol..

[13] Grogan S., Mechan J., Persson S., Finlay S., Hall M. (2019). “I’ve got a very dichotomous difference in the way that I perceive myself”: Positive and negative constructions of body image following cancer treatment. J. Health Psychol..

[14] Thornton M., Lewis-Smith H. (2023). “I listen to my body now”: A qualitative exploration of positive body image in breast cancer survivors. Psychol. Health.

[15] Policardo G.R., Nerini A., Di Gesto C., Matera C. (2024). The associations between positive body image, well-being and psychological flexibility in breast cancer survivors. Psychol. Health Med..

[16] Kvillemo P., Bränström R. (2014). Coping with breast cancer: A meta-analysis. PLoS ONE.

[17] Lazarus R.S., Folkman S. (1984). Stress, Appraisal, and Coping.

[18] Morris N., Moghaddam N., Tickle A., Biswas S. (2018). The relationship between coping style and psychological distress in people with head and neck cancer: A systematic review. Psycho-Oncology.

[19] Zhu H., Yang L., Yin H., Yuan X., Gu J., Yang Y. (2024). The influencing factors of psychosocial adaptation of cancer patients: A systematic review and meta-analysis. Health Serv. Insights.

[20] Cash T.F., Cash T.F., Pruzinsky T. (2002). Cognitive–behavioral perspectives on body image. Body Image: A Handbook of Theory, Research, and Clinical Practice.

[21] Zimet G.D., Dahlem N.W., Zimet S.G., Farley G.K. (1988). The Multidimensional Scale of Perceived Social Support. J. Pers. Assess..

[22] Maguire R., Hanly P., Maguire P. (2021). Living well with chronic illness: How social support, loneliness and psychological appraisals relate to well-being in a population-based European sample. J. Health Psychol..

[23] Cohen S., Wills T.A. (1985). Stress, social support, and the buffering hypothesis. Psychol. Bull..

[24] Belau M.H., Jung L., Maurer T., Obi N., Behrens S., Seibold P., Becher H., Chang-Claude J. (2024). Social relationships and their impact on health-related quality of life in a long-term breast cancer survivor cohort. Cancer.

[25] Cho H., Kang D., Shin D.W., Kim N., Lee S.K., Lee J.E., Nam S.J., Cho J. (2024). Social support during re-entry period and long-term quality of life in breast cancer survivors: A 10-year longitudinal cohort study. Qual. Life Res..

[26] Liu Y., Liu W., Ma Y., Yang X., Zhou H., Zhang T., Shao S. (2022). Research on body image cognition, social support and illness perception in breast cancer patients with different surgical methods. Front. Psychol..

[27] Zhang Y., Yan J., He H., Zhang L., Chen L., Li N., Li H., Zhang X., Zhang N., Sun S. (2024). The trajectories of psychosocial adjustment among young to middle-aged women with breast cancer: A prospective longitudinal study. Eur. J. Oncol. Nurs..

[28] Doori Z., Hekmat K., Mousavi P., Latifi S.M., Shahbazian H. (2022). Investigation of the relationship between perceived social support and body image in women with breast cancer. Middle East Curr. Psychiatry.

[29] Li Y., Li N., Wang J., Shang Q., Zhang B., Cao M. (2023). Effects of social support, family resilience, and individual resilience on fear of cancer recurrence among persons with breast cancer: A cross-sectional study. West. J. Nurs. Res..

[30] Bottaro R., Craparo G., Faraci P. (2023). What is the direction of the association between social support and coping in cancer patients? A systematic review. J. Health Psychol..

[31] Cohee A., Johns S.A., Alwine J.S., Talib T., Monahan P.O., Stump T.E., Cella D., Champion V.L. (2021). The mediating role of avoidant coping in the relationships between physical, psychological, and social well-being and distress in breast cancer survivors. Psycho-Oncology.

[32] Acoba E.F. (2024). Social support and mental health: The mediating role of perceived stress. Front. Psychol..

[33] Tylka T.L., Wood-Barcalow N.L. (2015). The Body Appreciation Scale-2: Item refinement and psychometric evaluation. Body Image.

[34] Lemoine J.E., Konradsen H., Lunde Jensen A., Roland-Lévy C., Ny P., Khalaf A., Torres S. (2018). Factor structure and psychometric properties of the Body Appreciation Scale-2 among adolescents and young adults in Danish, Portuguese, and Swedish. Body Image.

[35] Carver C.S. (1997). You want to measure coping but your protocol is too long: Consider the brief COPE. Int. J. Behav. Med..

[36] Pais-Ribeiro J.L., Rodrigues A.P. (2004). Questões acerca do coping: A propósito do estudo de adaptação do Brief COPE [Questions About Coping: Concerning the Adaptation Study of the Brief COPE]. Psicol. Saúde Doenças.

[37] Carvalho S., Pinto-Gouveia J., Pimentel P., Mala D., Mota-Pereira J. (2011). Características psicométricas da versão portuguesa da Escala Multidimensional de Suporte Social Percebido (Multidimensional Scale of Perceived Social Support—MSPSS) [Psychometric Characteristics of the Portuguese Version of the Multidimensional Scale of Perceived Social Support (MSPSS)]. Psychologica.

[38] Hayes A.F. (2017). Introduction to Mediation, Moderation, and Conditional Process Analysis.

[39] Adams R.N., Mosher C.E., Cohee A.A., Stump T.E., Monahan P.O., Sledge G.W., Champion V.L. (2017). Avoidant coping and self-efficacy mediate relationships between perceived social constraints and symptoms among long-term breast cancer survivors. Psycho-Oncology.

[40] Spatuzzi R., Vespa A., Lorenzi P., Miccinesi G., Ricciuti M., Cifarelli W., Susi M., Fabrizio T., Ferrari M.G., Ottaviani M. (2016). Evaluation of social support, quality of life, and body image in women with breast cancer. Breast Care.

[41] Scandurra C., Muzii B., La Rocca R., Di Bello F., Bottone M., Califano G., Longo N., Maldonato N.M., Mangiapia F. (2022). Social support mediates the relationship between body image distress and depressive symptoms in prostate cancer patients. Int. J. Environ. Res. Public Health.

[42] Cash T.F., Cash T.F. (2012). Cognitive-behavioral perspectives on body image. Encyclopedia of Body Image and Human Appearance.

